# Vanadium exposure and kidney markers in a pediatric population: a cross-sectional study

**DOI:** 10.1007/s00467-024-06561-9

**Published:** 2024-12-07

**Authors:** Elodia Rojas-Lima, Manolo Ortega-Romero, Octavio Gamaliel Aztatzi-Aguilar, Juan Carlos Rubio-Gutiérrez, Juana Narváez-Morales, Mariela Esparza-García, Pablo Méndez-Hernández, Mara Medeiros, Olivier Christophe Barbier

**Affiliations:** 1 Unidad de Investigación en Salud en El Trabajo, Centro Médico Nacional “Siglo XXI“, Instituto Mexicano Del Seguro Social (IMSS), Ciudad de Mexico, Mexico; 2 Consejo Nacional de Humanidades, Ciencias y Tecnologías (Conahcyt), Ciudad de Mexico, Mexico; 3 Departamento de Toxicología, Centro de Investigacio´n y de Estudios Avanzados del Instituto Politécnico Nacional, Ciudad de Mexico, Mexico; 4 Unidad de Investigación y Diagnóstico en Nefrología y Metabolismo Mineral Óseo, Hospital Infantil de México Federico Gómez, Ciudad de Mexico, Mexico; 5 Secretaría de Salud de Tlaxcala, Tlaxcala, Mexico; 6 Facultad de Ciencias de la Salud, Universidad Autónoma de Tlaxcala, Tlaxcala, Mexico; 7 Departamento de Farmacología, Facultad de Medicina, UNAM, Ciudad de Mexico, Mexico

**Keywords:** Vanadium, Kidney function, eGFR, Albuminuria, KIM-1, NGAL

## Abstract

**Background:**

Anthropogenic vanadium (V) emissions and exposure in the general population have recently increased. Experimental studies have shown that V is a nephrotoxic agent, but little is known about its effects on human kidney health. This work evaluated the association between urinary V concentrations with early kidney damage biomarkers and function in a pediatric population without any disease diagnosed.

**Methods:**

A cross-sectional study was carried out and included 914 healthy subjects and determined urinary V concentrations, glomerular filtration rate (eGFR), albumin–creatinine ratio (ACR), and the presence of kidney injury molecule 1 (KIM-1) and neutrophil gelatinase-associated lipocalin (NGAL) in urine. We evaluated the V effect using linear and logistic regression models adjusted by confounders.

**Results:**

Subjects found in the second and third tertiles of V showed an increase in urinary log-NGAL levels (βT2 vs. T1 = 0.39; 95% CI 0.14, 0.64, and βT3 vs. T1 = 1.04; 95% CI 0.75, 1.34) and log-KIM-1(βT2 vs. T1 = 0.25; 95% CI 0.04, 0.45 and βT3 vs. T1 = 0.39; 95% CI 0.15, 0.63); in addition, subjects in the third tertile had a positive and significant association with ACR (ORT3 vs. T1 = 1.96; 95% CI 1.29, 2.97) and increased in eGFR (βT3 vs. T1 = 3.98, 95% CI 0.39, 7.58), compared with subjects in the first tertile.

**Conclusions:**

Our study reports the effect of V on kidney markers in a healthy pediatric population. It could be related to tubulointerstitial lesions and function abnormalities.

**Graphical Abstract:**

**Figure:**
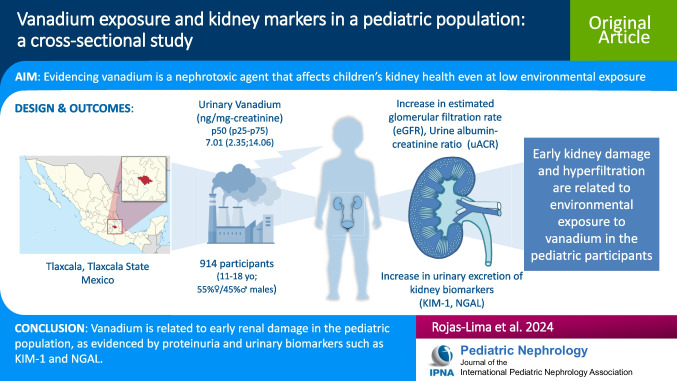
A higher resolution version of the Graphical abstract is available as Supplementary information.

**Supplementary Information:**

The online version contains supplementary material available at 10.1007/s00467-024-06561-9.

## Introduction

Vanadium (V) is a trace element widely distributed in nature and is found naturally in water and soil by erosion and weathering of rocks [1]. It has been recently debated whether there is a resurgence of V as an environmental risk due to the increased emissions in recent years [2]. Its demand in the steel industry and the production of batteries for renewable energy storage [3] has put it on the agenda of various regulatory agencies [4]. Its primary anthropogenic emissions result from burning fossil fuels and their use in industrial activities such as metallurgy to produce sulfuric acid, pigments, or coloring agents [1]. The highest atmospheric concentrations have been reported in urban areas, forming part of the particulate matter [5]. Consequently, inhalation is the main route of absorption, followed by gastrointestinal absorption due to consuming contaminated food [5].

Chronic kidney disease (CKD) represents a global and local public health problem. The global mortality rate from CKD increased by 41.5% between 1990 and 2017 [6]. In Mexico, it was 102.3% higher in the same period and the second highest cause of mortality in 2017 [7] and 2019 [8]. Tlaxcala, a central Mexican state, ranked second in national mortality in 2017 [7] and had the highest CKD mortality rate in people under 20 years of age in the period 2000 to 2014 [9], as well as CKD being the first mortality cause for all populations in 2019 [8].

CKD is a condition with a long evolution, generally occurring in the old adult population, and its main risk factors are diabetes, hypertension, and obesity [10]. In low- and middle-income countries, cases of Chronic Kidney Disease of unknown cause (CKDu) have been identified in the young adult population, which does not present traditional risk factors [11]. It is believed that exposure to food and water contaminated with multiple contaminants, including metals, could be related to the development of this condition [11, 12]; in this regard, a single cause has not been established. On the other hand, epidemiological studies have suggested that exposure to nephrotoxic metals, such as cadmium, arsenic, mercury, and lead, may increase the risk of CKD in the adult population [13]; however, other metals with nephrotoxic potential, like vanadium (V), have been little studied.

Experimental studies have demonstrated that V generates histological and physiological changes in several systems and organs, like respiratory, immune, renal, nervous, reproductive, and hematopoietic systems [14, 15]. The effects on the renal system include increased oxidative stress markers, inflammation, tubular cell damage, proteinuria, and hematuria [16].

In epidemiological studies, V exposure has been associated with cardiovascular, respiratory, and renal conditions. In a pooled analysis realized with European populations, long-term exposure to V in PM_2.5_ was associated with increased natural mortality [17]. High plasmatic V concentrations increased mortality for cardiovascular conditions [18], and urinary V levels were associated with hypertension prevalence in Chinese adults [19].

Kidney health studies are scarce and have contrasting results: no association between urinary V concentrations and estimated glomerular filtration rate (eGFR) [20, 21], positive association with CKD in adults [22, 23], pediatric populations [24], decline in kidney function [25], and distal renal tubular acidosis development [26]. On the other hand, our work group realized a pilot study in a population between 5 and 11 years of age in Apizaco, Tlaxcala state, Mexico, and found higher urinary concentrations of KIM-1, NGAL, and albumin–creatinine ratio (ACR) in the subjects with urinary V levels above the median in the study population [27].

Due to the scant information on the effect of V on kidney health in the pediatric population and the existence of possible damage mechanisms that have been reported in experimental studies, this report aimed to evaluate the association of urinary V levels with kidney health status through function and early damage biomarkers in a population of apparently healthy adolescents in the state of Tlaxcala.

## Materials and methods

### Study design and population

A cross-sectional study was carried out in 2019–2020 in three municipalities of Tlaxcala: Apetatitlán de Antonio Carvajal, Chiautempan, and Tlaxcala. The initial sample included 964 participants who were selected from public and private secondary and high schools under the following inclusion criteria: children and adolescents from 11 to 18 years of age, both sexes, without kidney disease diagnosis, with at least 1 year of residence in any municipality of the State of Tlaxcala, and who agreed to participate, having signed the letters of assent and informed consent. Fifty were eliminated because they did not have information on eGFR. This study was approved by the Ethics Committee of the Hospital Infantil de México Federico Gómez (HIM-2019–025) and by the Human Health Bioethics Committee (COBISH) of the Centro de Investigaciones y Estudios Avanzados del Instituto Politécnico Nacional (COBISH), -063/2020).

### Questionnaire

The sociodemographic characteristics, pathological personal history, hereditary family history, drinking and tobacco consumption habits, and environmental exposures of the participants and their parents were evaluated through a questionnaire. The socioeconomic level was measured with the information from the questionnaire with an approximation of the multidimensional measurement of poverty methodology for the Mexican population proposed by the Consejo Nacional de Evaluación de la Política de Desarrollo Social (CONEVAL, 2019).

### Anthropometrics and blood pressure

Weight, height, and abdominal circumference were measured. *Z*-scores for height, weight, and BMI were calculated using the STAT Growth Charts version 3.2 application that uses the National Center for Health Statistics 2000 Center for Disease Control Growth Data.

Blood pressure was measured in duplicate and at rest, and the subjects were subsequently classified according to the Clinical Practice Guideline for Screening and Management of High Blood Pressure in Children and Adolescents [28].

### Urine and blood sample collection

First-morning urine was obtained in sterile polypropylene containers, and peripheral venous blood samples were collected in an EDTA tube. Samples were centrifuged at 1000 rpm for 10 min, aliquoted into 1.5-mL tubes, transported on ice at 4 °C, and stored at –70 °C for further analysis.

### Determination of renal parameters

#### Serum creatinine and glomerular filtration rate determination

Serum creatinine was measured with the Dimension RxL Max Siemens equipment. The Bedside Schwartz formula was used to estimate the eGFR [29]. The experimental part was conducted at the Hospital Infantil de México Federico Gómez.

#### Urine albumin to creatinine ratio (ACR)

The ACR was estimated from the Randox Microalbumin (mALB)-immunoturbidimetric assay for albumin kit, and urinary creatinine was quantified with the CR510 kit (Randox Laboratories Ltd.) with the reaction of creatinine with alkaline picrate. With both determinations, the ACR indicator was estimated, and the following cut-off points were established according to the proteinuria classification: < 30 mg/g creatinine, ≥ 30 to < 300 mg/g creatinine, and ≥ 300 mg/g creatinine [30]. The limit of detection (LOD) value for ACR was 5.11 mg/L.

#### Early kidney damage biomarkers determination

The early kidney damage biomarkers chosen were kidney injury molecule-1 (KIM-1; MILLIPLEX MAP Human Kidney Injury Magnetic Bead Panel 1) and neutrophil gelatinase-associated lipocalin (NGAL; MILLIPLEX MAP Human Kidney Injury Magnetic Bead Panel 2). The LOD values were 0.05 ng/ml and 0.02 ng/ml for KIM-1 and NGAL, respectively. Early kidney damage biomarkers expressed in ng/ml were divided between urinary creatinine concentrations measured in mg/ml and reported in ng/mg creatinine.

The early kidney damage biomarkers, ACR, and urinary creatinine determinations were performed in the Toxicology laboratory in CINVESTAV.

### Urinary vanadium determinations

V determination was performed with inductively coupled plasma mass spectrometry (ICP-MS) using the Perkin Elmer NexION 300D model. The samples were nebulized and entered the plasma generated by the argon gas. The ions formed in the plasma were introduced into the mass analyzer (quadruples). They were classified according to their mass/charge ratio and directed to the simultaneous dual detector, which generates a signal proportional to the concentration of the element. An internal procedure was carried out to optimize the equipment, and it was verified that all the qualification parameters were within those established by the manufacturer. The elements were quantified using a validated method based on a calibration graph, evaluating duplicate samples for the elements, including blank samples and at least six different concentrations. As an analytical quality control, the accuracy and precision of the determination were evaluated, and the analytical variation coefficient was less than 10% in sample duplicates. For the accuracy evaluation, multi-element urine reference standards were used and analyzed with the study samples, obtaining a percentage of accuracy between 80 and 120%. The analysis was conducted at the Toxicology Research and Service Laboratory (LISTO) of the CINVESTAV Toxicology Department. The LOD value was 0.028 ng/ml. Urinary vanadium was divided between urinary creatinine concentrations and reported in ng/mg creatinine.

## Statistical analysis

### Early kidney damage biomarkers and vanadium imputation

The early kidney damage biomarkers and V found below the LOD were imputed with the iterative Markov Chains Monte Carlo (MCMC) data augmentation algorithm [31].

### Random missing values imputation

Identified variables with random missing values (between 5 and 30%) in the data set were imputed with the “missforest” algorithm, a non-parametric method for mixed variables [32].

### Descriptive analysis

Proportions were used to describe categorical variables. For continuous variables, normality was evaluated. Due to the data asymmetry, the general characteristics and urinary concentrations of early kidney damage biomarkers and V were analyzed through summary measures, such as median and percentiles.

### Bivariate analysis

Spearman’s correlations were performed to relate biomarkers of kidney damage and eGFR with urinary V concentrations. Additionally, urinary concentrations of early kidney damage markers, eGFR, ACR, and urinary V concentrations were compared in the strata of potential confounders with Spearman correlations for numerical variables and mean differences in categorical variables, with Mann–Whitney *U* test and Kruskal–Wallis test.

### Statistical modeling

To assess the association of biomarkers with urinary V concentrations, KIM-1 and NGAL were log-transformed, and V was classified in tertiles. The cut points were Tertile 1 ≤ 3.80; Tertile 2: 3.81 to 10.76; and Tertile 3: ≥ 10.77 according to the urinary levels in the study population. Multiple linear regression models were performed, with robust standard errors, due to heteroscedasticity in the residuals. Using logistic binary regression models to associate urinary V concentrations with the ACR was used; the ACR > 300 mg/g creatinine category was collapsed with ACR 30–300 mg/g creatinine category because the number of subjects in this stratum was *n* = 4. The adjustment variables were selected using two criteria: first, for a theoretical framework, if it is considered a risk factor in the development of kidney damage or being associated with V concentrations, and second, for statistical criteria. The final model retained the statistically significant variables or contributed to explaining the variance of the model. The trend test was evaluated using the median value of V, assigned to each tertile, and included in the model as a continuous variable. The values assigned were p50_T1_ = 0.99, p50_T2_ = 7.02, and p50_T3_ = 19.53 ng/mg creatinine. The assumptions of the models were evaluated. The marginal effects of V were estimated in log-NGAL, log-KIM, and eGFR and were reported as geometric means adjusted. The analyses were performed with STATA version 15 (StataCorp, College Station, TX, USA) and R software version 4.0.4.

## Results

The median age of the participants was 13 years (IQR = 12; 15); 55% were female, 18.71% were overweight, 12.58% were obese, 12.04% were born prematurely, and 8.86% had high blood pressure at the physical examination. 11.49% reported having been diagnosed by a physician with urinary tract infections, 0.98% with diabetes, 1.42% with hypertension, and 1.64% with renal disease (Table 1).

**Table 1:** Study population characteristics, renal biomarkers, and urinary vanadium concentrations

Characteristic	*n* = 914	
**Age (years), p50 (p25–p75)**	13	(12; 15)
**Sex, *n* %**		
Female	503	55.03
Male	411	44.97
**Body mass index,* n* %**		
Underweight	20	2.19
Normal	608	66.52
Overweight	171	18.71
Obesity	115	12.58
**Waist-to-height ratio, p50 (p25–p75)**	0.47	(0.44; 0.52)
**Premature birth (< 36 weeks gestational), *n* %**	110	12.04
**High blood pressure, *n* %**	81	8.86
**Personal pathological antecedents, *n* %**		
None	674	73.74
Diabetes	9	0.98
Arterial hypertension	13	1.42
Urinary tract infection	105	11.49
Renal disease	15	1.64
Others	93	10.18
Do not know	5	0.55
**Poverty**		
No	389	42.56
Moderate	459	50.22
Extreme	66	7.22
**Renal parameters**		
**eGFR (ml/min/ 1.73 m**^**2**^**), p50 (p25–p75)**	103.75	(92.04; 121.52)
**eGFR (ml/min/1.73 m**^**2**^**), *n* %**		
≥ 90	715	78.23
60–89	197	21.55
45–59	2	0.22
**Albumin–creatinine ratio (mg/g creatinine), p50 (p25–p75)**	5.08	(< LOD; 22.53)
**Albumin–creatinine ratio (mg/g creatinine), *n* %**		
< 30	742	81.18
30–300	168	18.38
> 300	4	0.44
**Early kidney damage biomarkers, p50 (p25–p75)**		
NGAL (ng/ml)	3.91	(1.24; 11.53)
NGAL (ng/mg creatinine)	4.48	(1.35; 13.05)
KIM-1 (ng/ml)	0.19	(0.07; 0.39)
KIM-1 (ng/mg creatinine)	0.21	(0.09; 0.46)
**Exposure biomarkers, p50 (p25–p75)**		
Vanadium (ng/ml)	7.52	(2.44; 11.62)
Vanadium (ng/mg creatinine)	7.01	(2.35; 14.06)

*p50*, 50^th^ percentile; *p25*, 25^th^ percentile; *p75*, 75^th^ percentile; *eGFR*, estimated glomerular filtration rate; *NGAL*, neutrophil gelatinase-associated lipocalin; *KIM-1*, kidney injury molecule 1; *LOD*, limit of detection

Regarding kidney parameters, the median eGFR was 103.75 ml/min/1.73 m^2^ (IQR 92.04; 121.52), and 18.88% presented proteinuria. The median NGAL value was 4.48 ng/mg creatine (IQR 1.35; 13.05), and the median KIM-1 value was 0.21 ng/mg creatinine (IQR 0.09; 0.46) (Table 1).

In bivariate analysis, the medians of NGAL, KIM-1, eGFR, and ACR in urinary V tertiles were significantly higher in the second and third tertile than in the first. Concerning kidney markers, compared with potential confounders, urinary concentrations of NGAL and eGFR were higher in females than males. Similarly, KIM-1 and eGFR were statistically different in age categories. The median ACR was higher only in the subjects in extreme poverty (p50 10.39 mg/g creatinine; IQR < LOD; 33.11) compared with the subjects in the non-poverty category (p50, 5.84 mg/g creatinine; IQR < LOD; 24.28) (Table 2).

**Table 2:** Median comparison between population characteristics with renal markers and urinary vanadium (*n* = 914)

Characteristic	NGAL(ng/mg creatinine)		KIM-1(ng/mg creatinine)		eGFR (ml/min/1.73 m^2^)		ACR (mg/g creatinine)		Vanadium (ng/mg creatinine)	
	**p50**	**(p25–p75)**	**p50**	**(p25–p75)**	**p50**	**(p25–p75)**	**p50**	**(p25–p75)**	**p50**	**(p25–p75)**
**Vanadium (ng/mg creatinine)**										
Tertile 1 (≤ 3.80)	**2.65**	(0.84; 8.83)*	0.17	(0.07; 0.39)*	100.02	(87.83; 115.93)*	2.06	(< LOD; 20.36)*	–	–
Tertile 2 (3.81 to 10.76)	**4.45**	(1.67; 10.52)*	0.23	(0.10; 0.44)*	103.94	(93.50; 117.78)*	5.38	(< LOD; 17.82)*	–	–
Tertile 3 (≥ 10.77)	**7.45**	(2.24; 24.03)*	0.26	(0.09; 0.63)*	110.015	(94.36; 127.92)*	7.76	(< LOD; 33.04)*	–	–
**Age (years)**										
10 to 12	**4.24**	(1.28; 14.60)	0.26	(0.10; 0.57)*	113.96	(98.56; 129.22)*	8.77	(< LOD; 25.17)	9.19	(4.44; 19.18)*
13 to 14	**4.40**	(1.29; 11.99)	0.21	(0.09; 0.43)*	106.98	(92.92; 124.71)*	4.93	(< LOD; 22.53)	8.86	(3.42; 16.73)*
15 to 18	**5.15**	(1.56; 13.40)	0.18	(0.07; 0.41)*	96.26	(85.74; 106.96)*	1.51	(< LOD; 19.71)	3.87	(0.69; 8.34)*
**Sex**										
Female	**6.67**	(2.38; 17.87)*	0.21	(0.09; 0.43)	**109.41**	(96.56; 126.38)*	5.30	(< LOD; 24.77)	7.03	(2.31; 14.41)
Male	**2.54**	(0.78; 8.49)*	0.22	(0.09; 0.49)	**97.39**	(87.7; 113.96)*	4.68	(< LOD; 21.23)	6.86	(2.42; 13.36)
**Body mass index**										
Underweight	6.60	(2.55; 36.27)	0.37	(0.10; 0.75)	114.73	(87.96; 122.71)	16.18	(0.99; 39.64)	7.02	(5.01; 24.26)
Normal	4.51	(1.26; 12.84)	0.22	(0.09; 0.47)	103.25	(91.19; 119.74)	4.68	(< LOD; 21.47)	6.66	(2.29; 13.64)
Overweight	4.77	(1.57; 11.49)	0.21	(0.09; 0.37)	105.46	(92.92; 121.69)	3.26	(< LOD; 21.06)	7.36	(2.26; 14.19)
Obesity	3.74	(1.33:14.67)	0.19	(0.08; 0.41)	105.79	(94.31; 126.38)	8.02	(< LOD; 27.18)	8.36	(2.84; 14.80)
**High blood pressure**										
Yes	4.202	(0.94; 12.33)	0.19	(0.06; 0.43)	101.92	(87.91; 114.31)	2.06	(< LOD; 14.11)	6.38	(1.87; 14.64)
No	4.519	(1.41; 13.05)	0.22	(0.09; 0.46)	104.27	(92.18; 121.65)	5.30	(< LOD; 22.64)	7.04	(2.42; 14.05)
**Personal pathological antecedents, *n* %**										
None	4.57	(1.36; 13.44)	0.21	(0.09; 0.47)	103.58	(92.32; 120.78)	4.94	(< LOD; 22.15)	6.86	(2.42; 14.14)
Diabetes	6.31	(1.55; 13.53)	0.23	(0.12; 0.41)	105.00	(89.78; 125.52)	6.37	(< LOD; 26.49)	6.67	(2.30; 12.91)
Arterial hypertension	4.45	(2.05; 16.28)	0.17	(0.10; 0.45)	108.72	(89.04; 128.29)	9.68	(< LOD; 38.74)	8.11	(1.18; 15.87)
Urinary tract infection	10.61	(1.57; 23.08)	0.26	(0.15; 0.33)	96.28	(93.37; 109.48)	4.98	(< LOD; 20.40)	4.47	(0.01; 15.99)
Renal disease	3.35	(3.03; 7.88)	0.16	(0.08; 0.35)	116.75	(102.96; 132.5)	3.81	(0.86; 4.15)	8.02	(1.67; 11.42)
Other	2.41	(0.90; 6.73)	0.26	(0.08; 0.46)	101.97	(87.22; 118.97)	2.88	(< LOD; 16.51)	7.77	(3.46; 11.97)
**Poverty**										
No	3.74	(1.26; 10.22)	0.25	(0.10; 0.45)	103.56	(90.27; 120.0)	**5.84**	(< LOD; 24.28)*	**5.25**	(1.93; 11.06)*
Moderate	5.11	(1.41; 13.98)	0.19	(0.08; 0.46)	102.62	(92.04; 121.65)	**3.60**	(< LOD; 18.15)*	**8.11**	(2.65; 16.73)*
Extreme	4.74	(1.27; 20.54)	0.22	(0.06; 0.57)	109.36	(96.37; 123.16)	**10.39**	(< LOD; 33.11)*	**10.21**	(5.14; 15.85)*

*p50*, 50^th^ percentile; *p25*, 25^th^ percentile; *p75*, 75^th^ percentile; *NGAL*, neutrophil gelatinase-associated Lipocalin; *KIM-1*, kidney injury molecule 1; *eGFR*, estimated glomerular filtration rate; *ACR*, albumin–creatinine ratio; *LOD*, limit of detection

*p < 0.05, Kruskal Wallis test for three or more categories, Mann–Whitney *U* test for two categories

Urinary V was higher in the subjects who were in extreme poverty (p50, 10.21; ng/mg creatinine; IQR 5.14; 15.85) compared with the subjects who were in the non-poor category (p50, 5.25 ng/mg creatinine; IQR 1.93; 11.06) (Table 2).

No significant differences were observed in renal markers and V, with pre-existing conditions of diabetes, arterial hypertension, urinary tract infections, renal disease, and other conditions (Table 2).

In multivariate analysis, model 3 was adjusted for age, sex, BMI, and poverty and observed that the subjects who were in the second and third tertile of V showed an increase in urinary concentrations of log-NGAL (β_T2.vs.T1._ = 0.39; 95% CI 0.14–0.64) and (β_T3.vs.T1._ = 1.06; 95% CI 0.77–1.35) with a significant p-trend, likewise for urinary levels of log-KIM-1(β_T2.vs.T1._ = 0.25; 95% CI 0.04–0.45) and (β_T3.vs.T1._ = 0.38; 95% CI 0.14–0.62) with a significant p-trend, compared with the subjects who were found in the first tertile (Table 3).

**Table 3:** Linear and logistic regression models for evaluating the association between vanadium urinary concentrations with early kidney damage biomarkers, glomerular filtration rate, and albuminuria

Biomarkers	log-NGAL (ng/mg creatinine)		log-KIM-1 (ng/mg creatinine)		eGFR (ml/min/1.73 m^2^)		ACR (mg/g creatinine)	
**Vanadium (ng/mg-creat.)**	***β***	**(95% CI)**	***β***	**(95% CI)**	***β***	**(95% CI)**	**OR**	**(95% CI)**
**Model 1**								
Tertile 1 (≤ 3.80)		Reference		Reference		Reference	1.00	Reference
Tertile 2 (3.81 to 10.76)	0.37	(0.11; 0.63)	0.28	(0.08; 0.48)	3.24	(− 0.03; 6.52)	0.77	(0.49; 1.21)
Tertile 3 (≥ 10.77)	0.96	(0.68; 1.25)	0.45	(0.22; 0.68)	9.44	(5.81; 13.08)	1.99	(1.34; 2.96)
log- Vanadium (ng/mg-creat.) (continuous**)**	0.17	(0.12; 0.23)	0.13	(0.08; 0.18)	1.12	(0.47;.1.77)	1.14	(1.03; 1.27)
*p-trend*		** < 0.001**		** < 0.001**		** < 0.001**		** < 0.001**
**Model 2**								
Tertile 1 (≤ 3.80)		Reference		Reference		Reference	1.00	Reference
Tertile 2 (3.81 to 10.76)	0.40	(0.16; 0.65)	0.25	(0.04; 0.45)	0.61	(− 2.42; 3.65)	0.76	(0.48; 1.19)
Tertile 3 (≥ 10.77)	1.06	(0.77; 1.35)	0.38	(0.14; 0.62)	4.09	(0.60; 7.58)	1.97	(1.30; 2.98)
log- Vanadium (ng/mg-creat.) (continuous**)**	0.20	(0.14; 0.25)	0.12	(0.07; 0.18)	0.23	(− 0.36; 0.83)	1.04	(1.02; 1.07)
*p-trend*		** < 0.001**		**0.005**		**0.015**		** < 0.001**
**Model 3**								
Tertile 1 (≤ 3.80)		Reference		Reference		Reference	1.00	Reference
Tertile 2 (3.81 to 10.76)	0.39	(0.14; 0.64)	0.25	(0.04; 0.45)	0.56	(− 2.51; 3.63)	0.74	(0.47; 1.17)
Tertile 3 (≥ 10.77)	1.04	(0.75; 1.34)	0.39	(0.15; 0.63)	3.98	(0.39; 7.58)	1.96	(1.29; 2.97)
log- Vanadium (ng/mg-creat.) (Continuous**)**	0.20	(0.14; 0.025)	0.12	(0.07; 0.18)	0.24	(− 0.35; 0.81)	1.04	(1.02; 1.07)
*p-trend*		** < 0.001**		**0.004**		**0.020**		** < 0.001**

Model 1: Crude; Model 2: Adjusted for age and sex; Model 3: Adjusted for age, sex, body mass index, and poverty

*log*, logarithm; *NGAL*, neutrophil gelatinase-associated lipocalin; *KIM-1*, kidney injury molecule 1; *eGFR*, estimated glomerular filtration rate; *ACR*, albumin/creatinine ratio; *CI*, confidence interval; *OR*, odds ratio

Concerning kidney function markers, the subjects in the third tertile of V were more likely to present proteinuria; the association was significant with ACR (OR_T3.vs. T1_. = 1.92; 95% CI 1.26–2.92) and an increase in the eGFR (β _T3.vs.T1_. = 4.04 95% CI 0.56–7.53) compared with subjects in the first tertile. Similar findings are observed in models 1, unadjusted, and 2, adjusted for age and sex (Table 3).

**Fig. 1:**
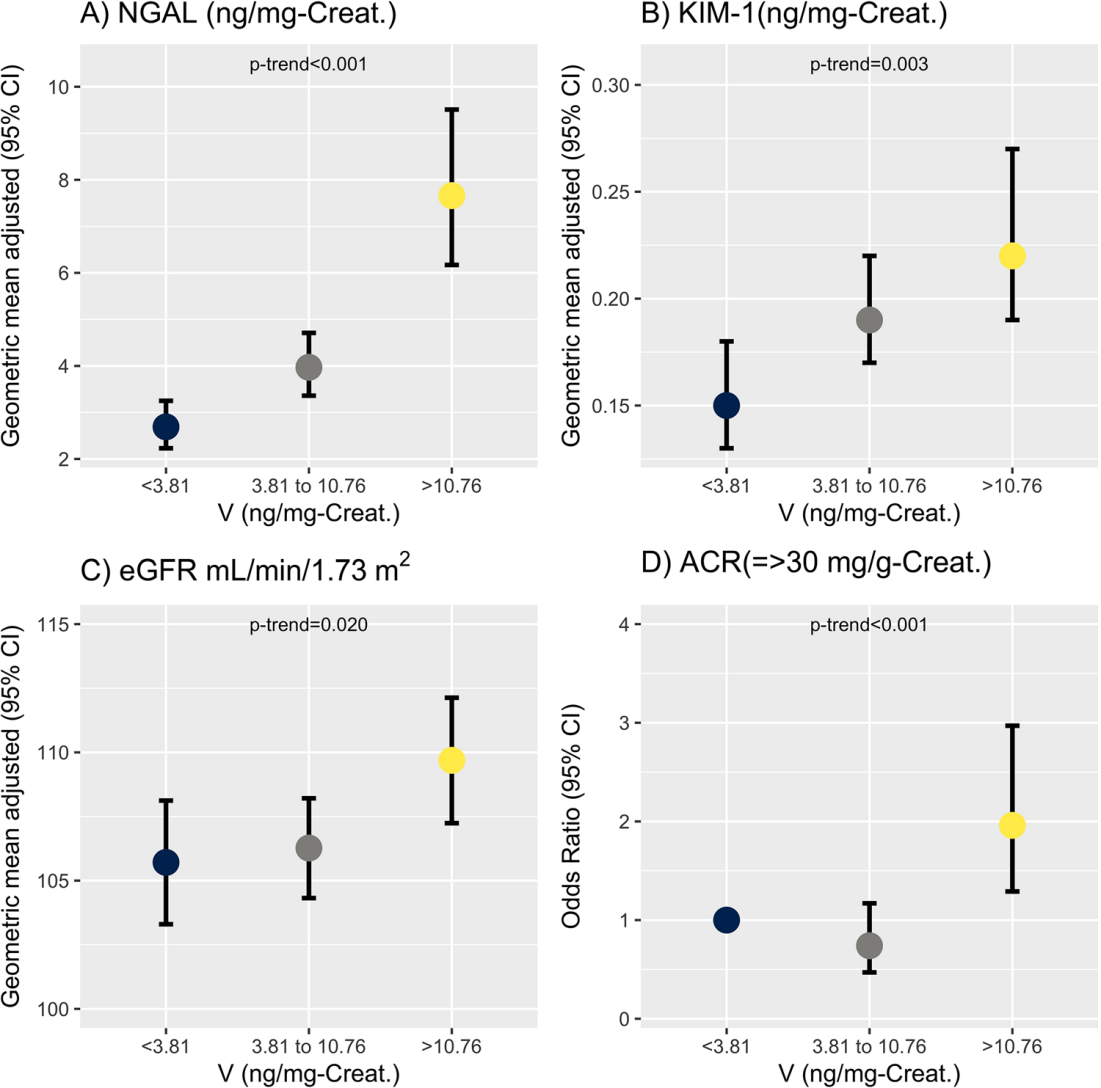
Adjusted geometric mean of early kidney damage biomarkers (**A** and **B**) and glomerular filtration rate (**C**) in tertiles of vanadium. The association of vanadium with albuminuria is represented as an odds ratio (**D**). The models to estimate the effects were adjusted for age, sex, body mass index, and poverty. Abbreviations: NGAL, neutrophil gelatinase-associated lipocalin; KIM-1, kidney injury molecule 1; eGFR, estimated glomerular filtration rate; ACR, albumin–creatinine ratio; CI, confidence interval; OR, odds ratio

Table 4 estimates the marginal effects of V on markers of kidney damage and the eGFR. The second and third tertiles of V show an increase in the geometric means of urinary concentrations of NGAL and KIM-1, as well as the eGFR, compared to the first tertile (Table 4 and Fig. 1).

**Table 4:** Geometric mean adjusted of early kidney damage biomarkers and glomerular filtration rate in vanadium tertiles

Description	NGAL (ng/mg creatinine)		KIM-1 (ng/mg creatinine)		eGFR (ml/min/1.73 m^2^)	
**Vanadium** **(ng/mg creatinine)**	**GM**^**a**^	**(95% CI)**	**GM**^**a**^	**(95% CI)**	**GM**^**a**^	**(95% CI)**
Tertile 1 (< 0.10 to 3.80)	2.69	(2.23; 3.25)	0.15	(0.13; 0.18)	105.71	(103.30; 108.12)
Tertile 2 (3.81 to 10.76)	3.97	(3.36; 4.71)	0.19	(0.17; 0.22)	106.27	(104.32; 108.21)
Tertile 3 (10.77 to 285.17)	7.66	(6.17; 9.51)	0.22	(0.19; 0.27)	109.69	(107.24; 112.13)

*NGAL*, neutrophil gelatinase-associated lipocalin; *KIM-1*, kidney injury molecule 1; *eGFR*, estimated glomerular filtration rate; *ACR*, albumin/creatinine ratio; *CI*, confidence interval; *OR*, odds ratio; *GM*, geometric mean

^a^Adjusted for age, sex, body mass index, and poverty

## Discussion

Our results suggest that urinary V is related to early kidney damage, evaluated with an increase in urinary concentrations of KIM-1 and NGAL, ACR presence over 30 mg/g creatinine, and an increase in eGFR; this latter being possibly secondary to a compensatory mechanism due to early nephron injuries. However, this is the first study that has evaluated the association of V with early kidney damage biomarkers and kidney function markers in an adolescent population without kidney alterations diagnosis in a geographical region with high mortality for CKD in the young population.

Epidemiological evidence on the effects of V on kidney function and CKD is scant and inconsistent. In a cross-sectional study conducted in China, which included older adults with diabetes, exposure to V has been associated with the possibility of developing CKD [22]; likewise, a longitudinal study on Chinese adults reported an association of V in plasma and decline in kidney function [25], while a longitudinal study conducted in middle-aged adults in Spain reported a negative and non-significant relationship between urinary V levels with eGFR and the presence of ACR [21]. Regarding kidney health, studies are scarce and have contrasting results. For example, in a study conducted in Taiwan in the adult population, no association was observed between urinary V concentrations and eGFR [20], similar to a longitudinal study in Spain [21]. Additionally, elevated V levels in soil were established as an environmental factor related to distal renal tubular acidosis development in northeastern Thailand [26]. In Sri Lanka, the regions with high V in soil were correlated with increased CKD cases [23]. A study in the pediatric population reported a rise in blood V levels in subjects with CKD due to environmental exposure and decreased glomerular filtration rate [24]. On the other hand, our work group realized a pilot study in a population between 5 and 11 years old in Apizaco, Tlaxcala state, Mexico, which found higher urinary concentrations of KIM-1, NGAL, and ACR in the subjects with urinary V levels above the median in the study population [27].

In this regard, and to contrast our results with these studies, we consider some essential points: These studies have been carried out in adult populations, where eGFR is lower in all populations than in ours due to a physiological process of decreased eGFR related to a reduction in creatinine clearance and its production for decreased muscular mass due to age [33]. On the other hand, the percentage of subjects in a condition of eGFR < 60 ml/min/1.73 m^2^ is higher compared with the rate of our population: 20.3% (*n* = 120) [22], and 8.8% (*n* = 126) [25], compared with 0.2% (*n* = 2), so it is not possible to assess the effect of V on this eGFR category in our study.

The findings in the alteration of kidney function, like the presence of ACR and the increase in markers of early kidney damage, suggest modifications in the initial stages, possibly in the proximal tubule, mainly because a decrease in eGFR is not observed [34].

When there is a decrease in the number of nephrons, molecular, functional, and structural changes are observed in the remaining nephrons as a compensatory mechanism [35]. They include cellular and tubular-glomerular hypertrophy, increased renal blood flow, eGFR, and protein levels [36]. These alterations increase solute delivery to healthy nephrons and the solute uptake rate by tubular epithelial cells [36]. These changes can lead to injury, sclerosis, and death of the nephrons. When compensatory changes are insufficient, a decrease in the filtration rate occurs. Due to the kidneys having the potential to suffer deterioration without clear symptomatic manifestations, the use of markers of early kidney damage is pertinent to identify early alterations and indicators of CKD manifestation [34].

This report included NGAL and KIM-1 determination, which were associated positively with the increase in urinary V concentration. Both biomarkers have been considered good predictors of CKD progression [37, 38]. NGAL is a protein highly modulated in various disease states; it is expressed by tubular epithelial cells as a kidney damage marker and released after tubular damage [39]. After a toxic kidney injury, significant upregulation of NGAL is noted in the kidney [40]. However, it has been reported that NGAL has an affinity for megalin and is reabsorbed in the proximal tubule by endocytosis [38]; therefore, the increase in this biomarker in urine may be related to alterations in the proximal segment of the tubule, suggesting that NGAL is a biomarker of kidney dysfunction [38]. In a normal kidney, KIM-1 is a membrane protein expressed in proximal tubule epithelial cells at low levels [41]. Its increase is associated with kidney fibrosis and inflammation in CKD and contributes to regenerating epithelial cells [42].

Only one study realized in the pediatric population in an age range between 5 and 11 years has evaluated the association of V with both early kidney damage biomarkers with similar findings to this report, where an increase in NGAL and KIM-1 urinary concentrations was observed in higher V urinary levels [27]. Additionally, both biomarkers have been evaluated in different populations exposed to other metals; for example, a study that included subjects from 17 to 67 years of age exposed to landfill emissions showed a positive relationship between blood levels of lead and urinary KIM-1 [40]. Similarly, KIM-1 was related to aluminum in Australian females over 50 [43]. In welding workers, NGAL and KIM-1 were related to exposure to aluminum, chromium, iron, nickel, and manganese, but only with NGAL [44]. On the other hand, in a cohort of Guatemalan sugarcane workers at risk for CKDu, NGAL was positively associated with higher urine cadmium [45]. In the pediatric Mexican population, KIM-1 was associated with chromium with a dose–response trend and with arsenic at the highest level of exposure [46].

The urinary V determination is considered the best exposure biomarker for this element [1]. Regarding the reference values, it has been established that for the occupational population exposed to V, urinary levels do not exceed 10 ng/ml [47]. However, our sample conformed to the pediatric population; 32.9% of the subjects had higher urinary values. Therefore, our data suggest kidney health effects may occur even at lower levels (4.02 to 9.85 ng/ml). Although the sources of V in this population have not been identified, it could be multiple sources due to its presence in particulate matter, industrial processes, the soil, and the consumption of foods with V levels [3]; nevertheless, this must be elucidated.

Oxidative stress has been considered a cause and effect in heavy metal-induced kidney toxicity. It plays a crucial role in the early process of glomerular and tubular damage in the kidney induced by heavy metals [12]. Experimental evidence suggests that the effects of V on the nephron occur by oxidative stress and play an essential role in tubular damage. A study in rats demonstrated that exposure to V by inhalation induced histological changes in the tubules, with signs of inflammation secondary to oxidative stress [16]. Another study also demonstrated V-induced lipid peroxidation, which is implicated in the toxic effects of V on the kidney [48]. In a hybrid mice model, after a subcutaneous application of oil rich in V, the development of glomerulonephritis was observed, with partial tubular and glomerular necrosis associated with kidney failure, as well as the development of arterial hypertension related to cellular dysfunction and disruption of the biochemical balance of electrolytes [49].

The prevalence of albuminuria in our study was higher than in other regions and countries. For example, in the USA, the prevalence was 13.15% in 1988–1994, 14.21% in 2003–2008, and 13.69% in 2009–2014 [50]. In our study, it was 18.82%. eGFR < 90 mil/min/1.73 m^2^ in our study was 22%, and in the USA, it was 31.46%, 39.31%, and 34.58% in the same period [50]. In Aguascalientes, another Mexican state, the prevalence of albuminuria was 8.52% [51]. Our group found a prevalence of urinary abnormalities and eGFR < 75 mL/min/1.73 m^2^ of 14.4% in a population between 6 and 15 years in Apizaco, Tlaxcala [52]. In contrast, in Lake Chapa Jalisco, the prevalence of albuminuria was 45.7% in a population between 1 and 17 years old [53].

Some methodological aspects and limitations must be considered when interpreting our results. The possibility of studying a biased sample is low because 94.8% of subjects accepting to participate were included in this report. A significant difference was found in the characteristics of included and non-included subjects only in age but not in V, NGAL, and KIM-1 values (Supplementary Table 1). The poverty indicator shown in this report was compared with the indicator reported by the CONEVAL in 2020 [54]. The CONEVAL poverty percentage reported for the state of Tlaxcala was 59.3% compared with 57.44% in our results. Regarding the extreme poverty indicator, the CONEVAL reported 9.8%, while in our results, this percentage was 7.22%. These findings suggest that the sample could adequately represent the target population.

Because the sample size in the ACR > 300 mg/g creatinine category (*n* = 4) was insufficient to assess its association with V, it was collapsed with the ACR 30–300 mg/g creatinine. To rule out any significant change in the estimators, we evaluated only the association of ACR 30–300 mg/g creatinine; we observed that the positive and significant association that we obtained with ACR ≥ 30 was preserved when eliminating from the analysis the four subjects with levels of ACR > 300 mg/g creatinine (data not shown).

A sensitivity analysis of the eGFR estimated with the pediatric Chronic Kidney Disease in Children under (age) 25 (CKiD U25) equation [55] and its association with V was performed. The results were similar in the association and significance in all models (Supplementary Table 2).

Other risk factors for developing CKD were explored, such as a history of urinary tract infections, diabetes, hypertension, or kidney disease at the time of the interview. We evaluated the behavior of the renal markers (NGAL, KIM-1, ACR, and eGFR) according to these conditions. We did not identify statistically significant differences between the healthy groups and those with a previous diagnosis of these diseases. Additionally, in the multivariate models, we included these variables in the adjustment and did not identify that they contributed to the association models of V with the renal markers, so they were not included in the final model (Supplementary Table 3).

Because 15 participants in the study population reported having some renal condition, we performed an additional analysis where these 15 participants were removed. The association results were preserved in direction and statistical significance (Supplementary Table 4).

Additionally, an analysis was carried out with the complete data set, assuming that the presence of missing data was random. In this regard, we observed that the associations between kidney function markers and V, with the results of this report and the complete data analysis, were similar, with the same direction of the association and the significance level (data not shown).

Due to the study design, it is not possible to establish temporality between the exposure and the event and discard reverse causality. In this regard, prospective studies are necessary to ensure the temporality between the exposure and the event.

Furthermore, it is essential to mention that the urinary concentrations of V in this study correspond to the determinations of total V, so we cannot establish which species of this element are present in our population. There are various oxidation states of V, but two main ones have been identified in organisms: V4 + and V5 + , with V5 + oxidative state being the most toxic since it is the most rapidly absorbed by erythrocytes [15]; it is the most reactive with numerous enzymes, and it is an inhibitor of Na + K + -ATPase of plasma membranes [1]. This could explain the inconsistencies in the results reported in the epidemiological studies since none of the studies report the speciation of V.

Additionally, it is necessary to evaluate the possibility that V is related to other potentially nephrotoxic elements with which it could interact, showing synergistic or antagonistic effects that this study does not report or measure. In this regard, it is essential to mention that there are multiple exposures to contaminants, including lead, arsenic, and cadmium, even at low doses, which must be considered to understand the effects observed in this population.

## Conclusions

In conclusion, despite the inconsistencies in epidemiological studies evaluating V and its association with kidney damage and the experimental evidence, our findings suggest that V is related to early kidney damage in the pediatric population and support the possible occurrence of damage in proximal tubular epithelial cells due to biomarkers such as KIM-1, NGAL, and the presence of proteinuria. As such, it is necessary to evaluate the evolution of kidney damage in prospective studies to confirm these results in this population.

## Supplementary Information

Below is the link to the electronic supplementary material.

Graphical abstract (PPTX 4140 KB)

Supplementary file1 (DOCX 16 KB)

Supplementary table 2 (DOCX 16.2 KB)

Supplementary table 3 (DOCX 20.2 KB)

Supplementary table 4 (DOCX 18.5 KB)

## Data Availability

Data will be made available on request.
