# Supplementary material for: Vanadium exposure and kidney markers in a pediatric population: a cross-sectional study
Source: Pediatr Nephrol. 2024 Dec 7;40(5):1689–700. doi: 10.1007/s00467-024-06561-9 (PMC11946968; doi:10.1007/s00467-024-06561-9)
Supplement: Supplementary file 1 — Graphical abstract (PPTX 4140 KB) [file 467_2024_6561_MOESM1_ESM.pptx]

## Slide 1
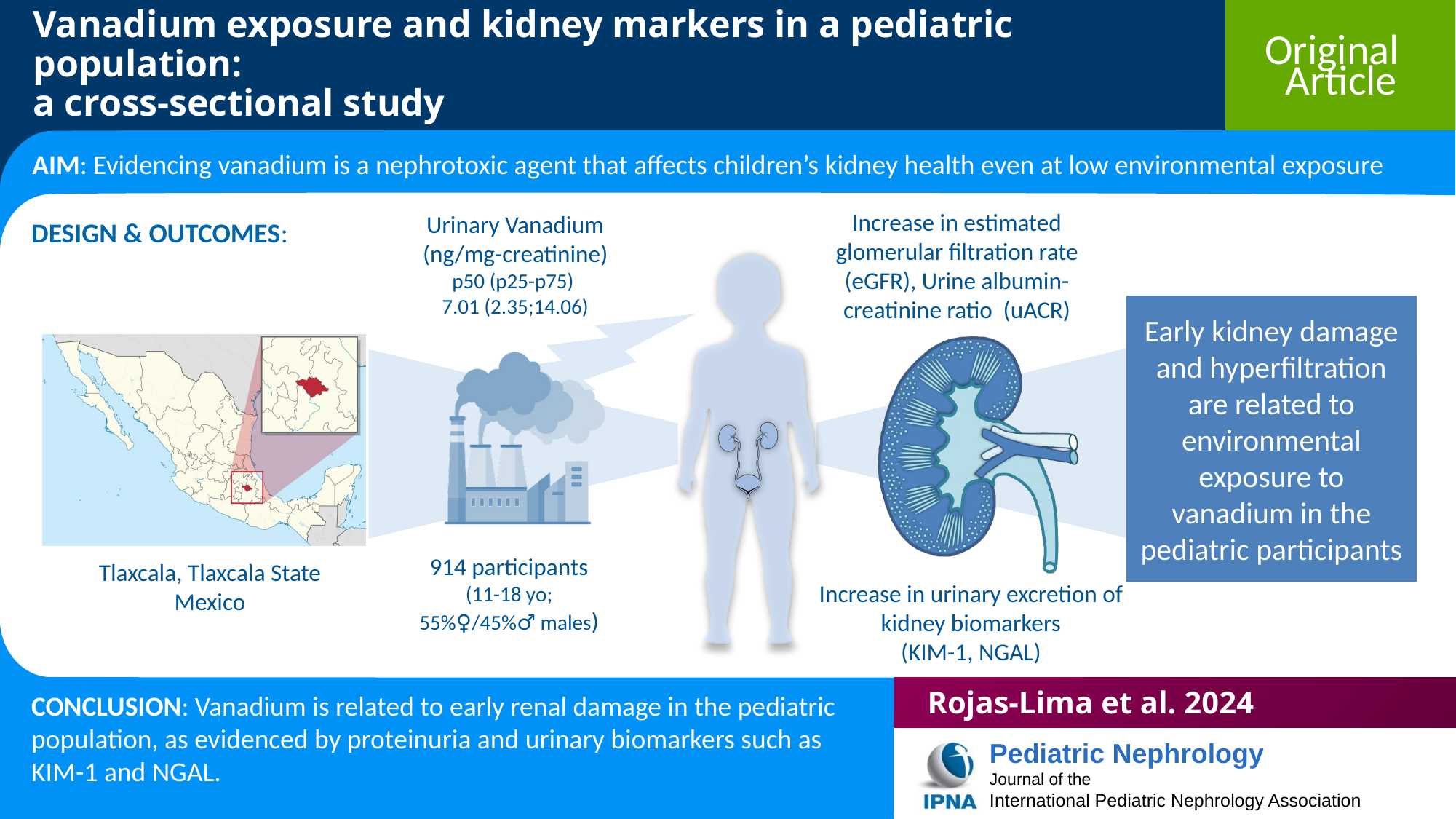

Vanadium exposure and kidney markers in a pediatric population:
a cross-sectional study
AIM: Evidencing vanadium is a nephrotoxic agent that affects children’s kidney health even at low environmental exposure
Increase in estimated glomerular filtration rate (eGFR), Urine albumin-creatinine ratio (uACR)
Urinary Vanadium (ng/mg-creatinine)
p50 (p25-p75)
7.01 (2.35;14.06)
DESIGN & OUTCOMES:
Early kidney damage and hyperfiltration are related to environmental exposure to vanadium in the pediatric participants
914 participants
(11-18 yo; 55%♀/45%♂ males)
Tlaxcala, Tlaxcala State Mexico
Increase in urinary excretion of kidney biomarkers
(KIM-1, NGAL)
Rojas-Lima et al. 2024
CONCLUSION: Vanadium is related to early renal damage in the pediatric population, as evidenced by proteinuria and urinary biomarkers such as KIM-1 and NGAL.
