# Supplementary material for: Vanadium exposure and kidney markers in a pediatric population: a cross-sectional study
Source: Pediatr Nephrol. 2024 Dec 7;40(5):1689–700. doi: 10.1007/s00467-024-06561-9 (PMC11946968; doi:10.1007/s00467-024-06561-9)
Supplement: Supplementary file 2 — Supplementary file1 (DOCX 16 KB) [file 467_2024_6561_MOESM2_ESM.docx]

| **Supplementary Table 1. Selected characteristics among included and non-included participants.** | | | | | | | |
| --- | --- | --- | --- | --- | --- | --- | --- |
|  | **Included** | | | **Non-included** | | |  |
| **Characteristic** | **n=914** | | | **n=50** | | | **p-value^a^** |
| **Age (years), p50 (p25-p75)** | 13 | (12;15) | | 15 | (13;16) | | <0.001 |
| **Sex, n %** |  |  | |  |  | |  |
| Female | 503 | 55.03 | | 29 | 58.00 | | 0.681 |
| Male | 411 | 44.97 | | 21 | 42.00 | |  |
| **BMI (kg/m2), p50 (p25-p75)** | 21.04 | (18.86;24.22) | | 21.16 | (18.5;22.4) | | 0.377 |
| **Renal parameters** |  |  | |  |  | |  |
| **Albumin-creatinine ratio (mg/g-creatinine), p50 (p25-p75)** | 5.08 | (<LOD; 22.53) | | <LOD | (<LOD; 22.53) | | 0.413 |
| **Early kidney damage biomarkers, p50 (p25-p75)** | | | |  |  | |  |
| NGAL (ng/ml) | 3.91 | (1.24;11.53) | | 3.01 | (1.08;7.57) | | 0.188 |
| NGAL (ng/mg-creatinine) | 4.48 | (1.35;13.05) | | 4.37 | (1.14;6.18) | | 0.375 |
| KIM-1 (ng/ml) | 0.19 | (0.07;0.39) | | 0.10 | (0.04;0.28) | | 0.105 |
| KIM-1 (ng/mg-creatinine) | 0.21 | (0.09;0.46) | | 0.13 | (0.05;0.58) | | 0.404 |
| **Exposure biomarkers, p50 (p25-p75)** | |  | |  |  | |  |
| Vanadium (ng/ml) | 7.52 | (2.44;11.62) | | 4.96 | (0.72; 14.38) | | 0.361 |
| Vanadium (ng/mg-creatinine) | 7.01 | (2.35;14.06) | | 6.39 | (0.84; 14.82) | | 0.533 |
| **Abbreviations:** p50, 50^th^ percentile; p25, 25^th^ percentile; p75, 75^th^ percentile; BMI, Body Mass Index; LOD, Limit of detection; NGAL, neutrophil gelatinase-associated Lipocalin; KIM-1, Kidney Injury Molecule 1; ^a^Mann Whitney U test; | | | | | | | |
|  |  | |  |  |  |  | |
