# Supplementary material for: Vanadium exposure and kidney markers in a pediatric population: a cross-sectional study
Source: Pediatr Nephrol. 2024 Dec 7;40(5):1689–700. doi: 10.1007/s00467-024-06561-9 (PMC11946968; doi:10.1007/s00467-024-06561-9)
Supplement: Supplementary file 3 — Supplementary table 2 (DOCX 16.2 KB) [file 467_2024_6561_MOESM3_ESM.docx]

| **Supplementary Table 2. Comparison of the vanadium association with the glomerular filtration rate estimated with two equations for the pediatric population (n=914)** | | | | | |
| --- | --- | --- | --- | --- | --- |
| **Biomarkers** | **eGFR Schwartz equation**  **(mil/min/1.73 m^2^ )** | | | **eGFR CKiD-U25 equation**  **(mil/min/1.73 m^2^ ))** | |
| **Vanadium (ng/mg-creat.)** | **β** | **(95% CI)** | **β** | | **(95% CI)** |
| **Model 1** |  |  |  | |  |
| Tertile 1 (≤3.80) |  | Reference |  | | Reference |
| Tertile 2 (3.81 to 10.76) | 3.24 | (-0.03; 6.52) | 2.16 | | (-1.01;5.34) |
| Tertile 3 (≥10.77 ) | 9.44 | (5.81;13.08) | 8.05 | | (4.54; 11.56) |
| log- Vanadium (ng/mg-creat.) (Continuous) | 1.12 | (0.47;.1.77) | 0.88 | | (0.26;1.50) |
| *p-trend* |  | **<0.001** |  | | **<0.001** |
| **Model 2** |  |  |  | |  |
| Tertile 1 (≤3.80) |  | Reference |  | | Reference |
| Tertile 2 (3.81 to 10.76) | 0.61 | ( -2.42; 3.65) | 0.73 | | (-2.45;3.92) |
| Tertile 3 (≥10.77 ) | 4.09 | (0.60; 7.58) | 5.05 | | (1.43; 8.67) |
| log- Vanadium (ng/mg-creat.). (Continuous) | 0.23 | ( -0.36;0.83) | 0.34 | | (-0.28;0.97) |
| *p-trend* |  | **0.015** |  | | **0.004** |
| **Model 3** |  |  |  | |  |
| Tertile 1 (≤3.80) |  | Reference |  | | Reference |
| Tertile 2 (3.81 to 10.76) | 0.56 | (-2.51;3.63) | 1.78 | | (1.16; 2.73) |
| Tertile 3 (≥10.77 ) | 3.98 | (0.39;7.58) | 5.20 | | (1.50;8.89) |
| log- Vanadium (ng/mg-creat.) (Continuous) | 0.24 | (-0.35;0.81) | 0.34 | | (-0.28;0.96)) |
| *p-trend* |  | **<0.020** |  | | **0.003** |

**Model 1**: Crude; **Model 2**: Adjusted for age and sex; **Model 3:** Adjusted for age, sex, Body Mass Index, and poverty. **Abbreviations:** log, logarithm; eGFR, estimated glomerular filtration rate; CI: confidence interval.
