# Supplementary material for: Vanadium exposure and kidney markers in a pediatric population: a cross-sectional study
Source: Pediatr Nephrol. 2024 Dec 7;40(5):1689–700. doi: 10.1007/s00467-024-06561-9 (PMC11946968; doi:10.1007/s00467-024-06561-9)
Supplement: Supplementary file 4 — Supplementary table 3 (DOCX 20.2 KB) [file 467_2024_6561_MOESM4_ESM.docx]

| **Supplementary Table 3. Linear and logistic regression models for evaluating the association between vanadium urinary concentrations with early kidney damage biomarkers, glomerular filtration rate, and albuminuria (n=914)** | | | | | | | | |
| --- | --- | --- | --- | --- | --- | --- | --- | --- |
| **Biomarkers** | **log-NGAL**  **(ng/mg-creatinine)** | | **log-KIM-1**  **(ng/mg-creatinine)** | | **eGFR**  **(mil/min/1.73 m^2^ )** | | **ACR**  **(mg/g-creatinine)** | |
| **Vanadium (ng/mg-creat.)** | **β** | **(95% CI)** | **β** | **(95% CI)** | **β** | **(95% CI)** | **OR** | **(95% CI)** |
| **Model 1** |  |  |  |  |  |  |  |  |
| Tertile 1 (≤3.80) |  | Reference |  | Reference |  | Reference | 1.00 | Reference |
| Tertile 2 (3.81 to 10.76) | 0.37 | (0.11;0.63) | 0.28 | (0.08;0.48) | 3.24 | (-0.03; 6.52) | 0.77 | (0.49;1.21) |
| Tertile 3 (≥10.77 ) | 0.96 | (0.68; 1.25) | 0.45 | (0.22;0.68) | 9.44 | (5.81;13.08) | 1.99 | (1.34; 2.96) |
| log- Vanadium (ng/mg-creat.) (Continuous) | 0.17 | (0.12;0.23) | 0.13 | (0.08;0.18) | 1.12 | (0.47;.1.77) | 1.14 | (1.03;1.27) |
| *p-trend* |  | **<0.001** |  | **<0.001** |  | **<0.001** |  | **<0.001** |
| **Model 2** |  |  |  |  |  |  |  |  |
| Tertile 1 (≤3.80) |  | Reference |  | Reference |  | Reference | 1.00 | Reference |
| Tertile 2 (3.81 to 10.76) | 0.40 | (0.16;0.65) | 0.25 | (0.04;0.45) | 0.61 | ( -2.42; 3.65) | 0.76 | (0.48;1.19) |
| Tertile 3 (≥10.77 ) | 1.06 | (0.77;1.35) | 0.38 | (0.14;0.62) | 4.09 | (0.60; 7.58) | 1.97 | (1.30; 2.98) |
| log- Vanadium (ng/mg-creat.) (Continuous) | 0.20 | (0.14;0.25) | 0.12 | (0.07;0.18) | 0.23 | ( -0.36;0.83) | 1.04 | (1.02;1.07) |
| *p-trend* |  | **<0.001** |  | **0.005** |  | **0.015** |  | **<0.001** |
| **Model 3** |  |  |  |  |  |  |  |  |
| Tertile 1 (≤3.80) |  | Reference |  | Reference |  | Reference | 1.00 | Reference |
| Tertile 2 (3.81 to 10.76) | 0.39 | (0.14;0.64) | 0.25 | (0.04;0.45) | 0.56 | (-2.51;3.63) | 0.74 | (0.47; 1.17) |
| Tertile 3 (≥10.77 ) | 1.04 | (0.75;1.34) | 0.39 | (0.15;0.63) | 3.98 | (0.39;7.58) | 1.96 | (1.29; 2.97) |
| log- Vanadium (ng/mg-creat.) (Continuous) | 0.20 | (0.14;0.025) | 0.12 | (0.07;0.18) | 0.24 | (-0.35;0.81) | 1.04 | (1.02;1.07) |
| *p-trend* |  | **<0.001** |  | **0.004** |  | **0.020** |  | **<0.001** |
| ***Model 4*** |  |  |  |  |  |  |  |  |
| Tertile 1 (≤3.80) |  | Reference |  | Reference |  | Reference | 1.00 | Reference |
| Tertile 2 (3.81 to 10.76) | 0.42 | (0.17;0.67) | 0.25 | (0.42;0.45) | 0.63 | (-2.45;3.70) | 0.79 | (0.50;1.25) |
| Tertile 3 (≥10.77 ) | *1.05* | (0.17;0.67) | 0.38 | (0.14;0.62) | 3.91 | (0.36;7.45) | 1.90 | (1.23;1.24) |
| Continuous log- Vanadium (ng/mg-creat.) (Continuous) | *0.20* | **(0.15;0.26)** | 0.12 | (0.07;0.18) | 0.23 | (-0.35;0.82) | 1.14 | (1.02;1.26) |
| *p-trend* |  | **<0.001** |  | **0.004** |  | **0.021** |  | **<0.001** |
| **Model 1**: Crude; **Model 2**: Adjusted for age and sex; **Model 3:** Adjusted for age, sex, Body Mass Index, and poverty; **Model 4:** Adjusted for age, sex, Body Mass Index, poverty, and personal pathological antecedents (diabetes, arterial hypertension, urinary tract infections, renal disease).  **Abbreviations:** log, logarithm; NGAL, neutrophil gelatinase-associated Lipocalin; KIM-1, Kidney Injury Molecule 1; eGFR, estimated glomerular filtration rate; ACR, albumin/creatinine ratio; CI: confidence interval; OR, odds ratio. | | | | | | | | |
