# Supplementary material for: Vanadium exposure and kidney markers in a pediatric population: a cross-sectional study
Source: Pediatr Nephrol. 2024 Dec 7;40(5):1689–700. doi: 10.1007/s00467-024-06561-9 (PMC11946968; doi:10.1007/s00467-024-06561-9)
Supplement: Supplementary file 5 — Supplementary table 4 (DOCX 18.5 KB) [file 467_2024_6561_MOESM5_ESM.docx]

| **Supplementary Table 4. Linear and logistic regression models for evaluating the association between vanadium urinary concentrations with early kidney damage biomarkers, glomerular filtration rate, and albuminuria.**  **Sensitivity analysis excluded pre-existent renal conditions participants (n=899).** | | | | | | | | |
| --- | --- | --- | --- | --- | --- | --- | --- | --- |
| **Biomarkers** | **log-NGAL**  **(ng/mg-creatinine)** | | **log-KIM-1**  **(ng/mg-creatinine)** | | **eGFR**  **(mil/min/1.73 m^2^ )** | | **ACR**  **(mg/g-creatinine)** | |
| **Vanadium (ng/mg-creat.)** | **β** | **(95% CI)** | **β** | **(95% CI)** | **β** | **(95% CI)** | **OR** | **(95% CI)** |
| **Model 1** |  |  |  |  |  |  |  |  |
| Tertile 1 (≤3.80) |  | Reference |  | Reference |  | Reference | 1.00 | Reference |
| Tertile 2 (3.81 to 10.76) | 0.36 | (0.10;0.62) | 0.28 | (0.08;0.49) | 3.05 | (-0.26; 6.35) | 0.81 | (0.51;1.27) |
| Tertile 3 (≥10.77 ) | 0.96 | (0.67; 1.25) | 0.44 | (0.21;0.67) | 9.54 | (5.87;13.21) | 1.86 | (1.24; 2.77) |
| log- Vanadium (ng/mg-creat.) (Continuous) | 0.17 | (0.12;0.23) | 0.13 | (0.08;0.18) | 1.13 | (0.46;1.79) | 1.13 | (1.02;1.26) |
| *p-trend* |  | **<0.001** |  | **<0.001** |  | **<0.001** |  | **<0.001** |
| **Model 2** |  |  |  |  |  |  |  |  |
| Tertile 1 (≤3.80) |  | Reference |  | Reference |  | Reference | 1.00 | Reference |
| Tertile 2 (3.81 to 10.76) | 0.39 | (0.14;0.64) | 0.25 | (0.04;0.45) | 0.37 | ( -2.69; 3.44) | 0.78 | (0.49;1.22) |
| Tertile 3 (≥10.77 ) | 1.05 | (0.76;1.34) | 0.36 | (0.12;0.60) | 3.93 | (0.41; 7.45) | 1.74 | (1.15; 2.65) |
| log- Vanadium (ng/mg-creat.) (Continuous) | 0.20 | (0.14;0.26) | 0.12 | (0.07;0.18) | 0.24 | ( -0.36;0.83) | 1.12 | (1.01;1.24) |
| *p-trend* |  | **<0.001** |  | **0.004** |  | **0.015** |  | **<0.001** |
| **Model 3** |  |  |  |  |  |  |  |  |
| Tertile 1 (≤3.80) |  | Reference |  | Reference |  | Reference | 1.00 | Reference |
| Tertile 2 (3.81 to 10.76) | 0.37 | (0.12;0.62) | 0.25 | (0.04;0.45) | 0.29 | (-2.78;3.37) | 0.77 | (0.48; 1.21) |
| Tertile 3 (≥10.77 ) | 1.03 | (0.74;1.32) | 0.37 | (0.13;0.61) | 3.94 | (0.37;7.52) | 1.78 | (1.16; 2.73) |
| log- Vanadium (ng/mg-creat.) (Continuous) | 0.20 | (0.14;0.25) | 0.12 | (0.07;0.18) | 0.24 | (-0.35;0.83) | 1.12 | (1.01;1.24) |
| *p-trend* |  | **<0.001** |  | **0.003** |  | **0.020** |  | **<0.001** |
| **Model 1**: Crude; **Model 2**: Adjusted for age and sex; **Model 3:** Adjusted for age, sex, body mass index, and poverty. **Abbreviations:** log, logarithm; NGAL, neutrophil gelatinase-associated Lipocalin; KIM-1, Kidney Injury Molecule 1; eGFR, estimated glomerular filtration rate; ACR, albumin/creatinine ratio; CI: confidence interval; OR, odds ratio. | | | | | | | | |
